# Households' Food Insecurity and Its Association with Demographic and Socioeconomic Factors in Gaza Strip, Palestine: A Cross-Sectional Study

**DOI:** 10.4314/ejhs.v32i2.18

**Published:** 2022-03

**Authors:** Abdel Hamid El Bilbeisi, Ayoub Al-Jawaldeh, Ali Albelbeisi, Samer Abuzerr, Ibrahim Elmadfa, Lara Nasreddine

**Affiliations:** 1 Department of Nutrition, School of Medicine and Health Sciences, University of Palestine, Gaza Strip, Palestine; 2 Regional Office for the Eastern Mediterranean (EMRO), World Health Organization (WHO), Cairo, Egypt; 3 Health Research Unit, Palestinian Ministry of Health; 4 Visiting Scholar with the School of Public Health, Department of Social and Preventive Medicine, University of Montreal, Montréal, QC, Canada; 5 Department of Nutrition, Faculty of Life Sciences, University of Vienna, Vienna, Austria; 6 Nutrition and Food Sciences Department, Faculty of Agriculture and Food Sciences, American University of Beirut, Beirut, Lebanon

**Keywords:** Demographic factors, Food insecurity, Gaza strip, Households, Socioeconomic factors

## Abstract

**Background:**

This sudy aimed to identify the prevalence of household's food insecurity and its association with demographic and socioeconomic factors.

**Methods:**

A cross-sectional study was conducted in September 2021 among a representative sample of households in the Gaza strip governorates. A total of 1167 households randomly selected from all five governorates and were included in the study. The Radimer/Cornell food security scale was used to determine the prevalence and levels of household food insecurity. The household's demographic and socioeconomic characteristics were obtained using an interview-based questionnaire. Statistical analysis was performed using SPSS version 25.

**Results:**

The overall prevalence of household's food insecurity was 71.5%. The prevalence by governorates was highest in Gaza (30.8%), followed by Khanyounis (23.0%), North-Gaza (18.6%), Middle-Area (15.2%) and Rafah (12.4%). Regarding the food insecurity levels, 333 (28.5%) of the households were food secure, 422 (36.2%) had mild food insecurity, 161 (13.8%) had moderate food insecurity, and 251 (21.5%) had severe food insecurity. Significant associations were found between governorates, monthly income, homeownership, work status with the household's food insecurity, (Crude OR [COR] = 2.02, 95% CI = [1.02–3.98], P value < 0.05), (COR = 2.00, 95% CI = [1.04–2.75], P value < 0.05), (COR = 2.36, 95% CI = [1.39–3.99], P value < 0.05), and (COR = 1.14, 95% CI = [0.66–1.97], P value < 0.05), respectively.

**Conclusions:**

Our study demonstrates that food insecurity is highly prevalent in the Gaza strip and is associated with poor living conditions. Therefore, this high prevalence should be seriously discussed and urgently considered.

## Introduction

Since 2014, the global incidence of moderate or severe food insecurity has steadily increased, with the projected increase in 2020 equaling the preceding five years combined (1). In 2020, over one-third of the world's population lacked enough food, a rise of nearly 320 million people in only one year (2). In 2020, over 12% of the world population, or 928 million people, were extremely food insecure, up 148 million from 2019 (3). Healthy diets are out of reach for roughly 3 billion people, mainly the poor, in every area of the world in 2019, due to the high expense of healthy meals combined with consistently high levels of economic disparity (4). Conflicts, climatic variability and extremes, and economic slowdowns and downturns (now worsened by the COVID-19 pandemic) are all major food insecurity and malnutrition drivers increasing in frequency and intensity and increasingly occurring in combination (3).

The Gaza strip already suffers from a prolonged economic deterioration caused by more than 15 years of siege and sustained undermining of potential economic rehabilitation. Fifty-three percent of individuals in the Gaza strip were poor in 2017 (5). According to World Bank preliminary estimates, the percentage of poor households will increase to 64% in Gaza due to the COVID-19 outbreak (6). Prior to the COVID-19 outbreak, the Palestinian situation was one in which war remained the primary cause of food insecurity, affecting the lives of 1.7 million people and exacerbated by high poverty and unemployment rates. In early 2021, this number was expected to rise to two million (7). These factors led to a significant increase in vulnerability and food insecurity among the households in the Gaza strip (5, 8).

Food security refers to a scenario in which all people have physical, social, and economic access to enough, safe, and nutritious food to fulfil their dietary needs and food choices for an active and healthy life at all times (9). This definition includes four dimensions (availability, access, utilization and stability), which are integral to achieving food security (10, 11). In the present study, the 10-item Radimer/Cornell food security scale was used for determining the prevalence and levels of a household's food insecurity (12, 13). Past research shows that the scale is a viable and reliable tool for measuring household food insecurity in a culturally diverse setting (14–16).

The demand for measuring the prevalence and levels of household's food insecurity in the Gaza strip is high due to the constantly deteriorating situations. Policy-makers need it, researchers and program officers trying to target, monitor and evaluate interventions and policy measures to improve food security. The present study was conducted to determine the prevalence of household's food insecurity and its association with demographic and socioeconomic factors in the Gaza Strip, Palestine.

## Materials and Methods

**Study design**: This cross-sectional, community-based study was conducted in September 2021 among a representative sample of households in the Gaza strip governorates. Random selection was employed to identify the eligible households after applying the inclusion and exclusion criteria.

**Study location**: The current study was conducted in the households of the Gaza strip, Palestine. The estimated population of the Gaza strip is about 2,106,745 million (17). The Gaza strip is divided into five governorates: North-Gaza, Gaza, Middle-Area, Khanyounis, and Rafah (18).

**Sample size**: The sample size was calculated to determine the number of households that were involved in the current study using the single proportion formula (19), where the prevalence of household food insecurity in the Gaza strip = 54% (20). The calculated sample size was 1060.2, to which we added 10% as an expected nonresponse rate. Therefore, the final sample size was 1060.2 + 10% (106) = 1166.2. A total of 1167 households were selected from all governorates based on their population density and were distributed in each governorate as follows 225 from North Gaza, 408 from Gaza, 168 from the Middle-Area, 223 from Khanyounis, and 143 from Rafah governorate.

**Sampling technique**: A list of households was taken from the municipal office in each governorate. Every ten households were screened for eligibility criteria. The household, which did not meet the eligibility criteria or a head of household refused to participate were excluded, and the next household was selected. A purposive selection (proportionally based on the population density in each governorate) was conducted from the selected household utilizing eligibility criteria.

**Eligibility issue**: Households having at least one child (male or female), aged less than 18 years, and living with his/her mother in the same household were included in the present study. In addition, households that do not have children aged less than 18 years, pregnant mothers in second and third trimesters, and fathers with disabilities or chronic disease were excluded from the present study.

## Data Collection

**Interview questionnaire**: An interview-based questionnaire was used to collect information about households' food insecurity and demographic, socioeconomic characteristics of the 1167 selected households. The data was collected from the head of the household (mothers or fathers) by ten qualified data collectors who were trained and prepared by the researcher. A thirty-household pilot study was conducted to allow the researcher to test the study's instruments. The questionnaire and data collecting procedure were modified/adjusted based on the results of the pilot study's findings.

**Food insecurity measurement**: In the present study, the 10-items Radimer/Cornell food security scale was used for determining the prevalence and levels of a household's food insecurity (12). Levels of food insecurity of the households were classified as follows as follows: 1) Food secure: Negative answers to all hunger and food insecurity items; 2) Household food insecure (mild food insecurity): Positive answers ('sometimes true’ or ‘often true') to one or more household-level item(s) (1–4) but not to adult or child-level items; 3) Individual food insecure (moderate food insecurity): Positive answers to one or more adult-level item(s) (5–7) or the item about the quality of children's diet (8), but not to items about the quantity of childrens' intake (9–10); and Child hunger (severe food insecurity): Positive answers to items about the quantity of children's intake (9–10) (13).

**Demographic and socioeconomic characteristics**: Individual face-to-face interview with the heads of households (mothers or fathers) was conducted to collect the demographic and socioeconomic characteristics of the selected households.

**Ethical approval**: The study protocol was approved by the Palestinian Health Research Council (Helsinki Ethical Committee of Research Number: PHRC/HC/961/21), University of Palestine Ethical Committee of Research, the Palestinian Ministry of Health, and Ministry of Interior. Furthermore, informed consent was obtained from each participant.

**Data analysis**: The Statistical Package for Social Science (SPSS) for Windows (version 25) was used for data analysis. Descriptive statistics were used to describe continuous and categorical variables. The univariate logistic regression model was applied to determine the association between a household's food insecurity prevalence and demographic and socioeconomic variables. A P-value less than 0.05 was considered statistically significant.

## Results

A total of 1167 households from the Gaza strip governorates were included in the current study. The results show that 67.2% of surveyed households' heads were males, and 40% were females. The mean age (years) of the study participants was 38.70±11.39. More than half of the study participants (53.8%) were university graduates, 40.5% were unemployed, and 14.4% were living in rented homes. In addition, 44.3%, 36.6%, and 19.1% of the surveyed households were located in Gaza strip cities, refugee camps, and villages, respectively. The mean of family members of the included households was 5.83±2.83. Furthermore, more than half of the surveyed households (52.2%) earn a monthly average income of less than 1000 New Israeli Shekel (NIS), equivalent to almost 309.21 USD; and about 59.2% of the households receive food aid (Table 1).

**Table 1:** Demographic and socioeconomic characteristics of the included households

Variables	Number of respondents (n=1167)	Percentage (%)	Mean±SD
**Proxy gender**			
Male	784	67.2	
Female	383	32.8	
**Marital status**			
Single	0.00	0.00	
Married	1080	92.5	
Divorced	39.0	3.40	
Widowed	48.0	4.10	
**Age (years)**			38.70±11.39
**Educational level**			
Illiterate	30.0	2.50	
Primary	31.0	2.70	
Preparatory	136	11.7	
Secondary	341	29.3	
University	629	53.8	
**Work status**			
Have work	694	59.5	
Do not have work	473	40.5	
**Governorates**			
North Gaza	225	19.2	
Gaza	408	35.0	
Middle-Area	168	14.4	
Khanyounis	223	19.1	
Rafah	143	12.3	
**Homeownership**			
Owned	999	85.6	
Rented	168	14.4	
**Living area**			
City	517	44.3	
Village	223	19.1	
Camp	427	36.6	
**Family members**			5.83±2.83
**Monthly income (NIS)**			
Less than 1000	610	52.2	
1000 to 2000	369	31.6	
2001 to 3000	136	11.7	
More than 3000	52.0	4.50	
**Receive food aid**			
Yes	691	59.2	
No	476	40.8	

Data are expressed as means ± SD for continuous variables and as percentages for categorical variables. NIS: New Israeli Shekel

Regarding food insecurity at the household level, Table 2 shows that 63.4% of the study participants expressed their concerns about running out of food or raw materials for cooking before getting money to buy it. In addition, 55.7% of participants reported that they always eat the same thing for several days in a row because they only have a few different kinds of food on hand and do not have money to buy more. Regarding food insecurity at the adult individual level, Table 2 demonstrates that 36.6% conveyed that they are often hungry but do not eat because they do not have enough money to buy food; and 41.7% stated that they were not able to eat properly or eat to satiety because they do not have enough money to buy food. Furthermore, as regards food insecurity at the individual child level, 56% of study participants indicated that they could not provide a balanced meal to their children because they did not have enough money to provide food; and 35.3% reported that they know sometimes their children are hungry. Still, they cannot do anything because they cannot purchase food more than what they always buy.

**Table 2:** Households food insecurity measurements based on Radimer/Cornell food security scale (n =1167)

No.	Questions	Not true n (%)	Sometimes true n (%)	Always true n (%)	Mean±SD	Weighted mean (%)
	**A. Household-level**					
**1.**	I worry that if the food or raw materials for cooking will run out before I could have more money to buy food.	427 (36.6)	552 (47.3)	188 (16.1)	2.20±0.69	73.3
**2.**	Food or raw materials for cooking that I bought for my family at home is always run out fast, and I do not have the money to repurchase food.	605 (51.8)	432 (37.0)	130 (11.2)	2.40±0.68	80.0
**3.**	I did not have enough food or raw materials for cooking or preparing a family meal (for the tomb of the morning, noon or night), and I did not have enough money to buy food.	633 (54.2)	413 (35.4)	121 (10.4)	2.43±0.67	81.0
**4.**	We eat the same thing for several days in a row because we only have a few different kinds of food on hand and do not have money to buy more.	517 (44.3)	505 (43.3)	145 (12.4)	2.31±0.68	77.0
	**B. Individual-level (Adult)**					
**5.**	I am often hungry, but I do not eat because I do not have enough money to buy food.	743 (63.7)	345 (29.5)	79.0 (6.80)	2.56±0.61	85.3
**6.**	I only eat a little of what should I eat because I don't have enough money to buy food.	654 (56.0)	416 (35.6)	97.0 (8.40)	2.47±0.64	82.3
**7.**	I could not eat properly or eat to satiety because I did not have enough money to buy food.	681 (58.3)	385 (33.0)	101 (8.70)	2.49±0.65	83.0
	**C. Individual-level (Child)**					
**8.**	I cannot provide a balanced meal to my children because I do not have enough money to provide food.	513 (44.0)	477 (40.9)	177 (15.1)	2.28±0.71	76.0
**9.**	My children do not eat enough or always lack food because I cannot buy enough food.	682 (58.4)	394 (33.8)	91 (7.80)	2.50±0.63	83.3
**10.**	Sometimes my children are hungry, but I cannot do anything because I cannot buy food over what I always purchase.	755 (64.7)	324 (27.8)	88 (7.50)	2.57±0.62	85.6

Data are expressed as percentages for categorical variables

Table 3 shows the household classification according to food security status and the levels of household food insecurity. Overall, most of the included households, 834 (71.5%), were food insecure, and only 333 (28.5%) were food secure. In addition, 333 (28.5%) of the included households were food secure, 422 (36.2%) had households food insecurity (mild food insecurity), 161 (13.8%) had individual food insecurity (moderate food insecurity), and 251 (21.5%) had a child hunger (severe food insecurity).

**Table 3:** Classification of households according to food security status and household food insecurity levels in the Gaza strip

Characteristics	Frequency (n=1167)	Percentage (%)
**Overall food security status**		
1. Households food secure	333	28.5
2. Households food insecure	834	71.5
**Levels of households food insecurity**		
1. Households food secure	333	28.5
2. Households food insecure (mild food insecurity)	422	36.2
3. Individual food insecure (moderate food insecurity)	161	13.8
4. Child hunger (severe food insecurity)	251	21.5

Data are expressed as percentages for different categorical variables. The household's food insecurity levels were based on the Radimer/Cornell food security scale (13).

**Figure 1:**
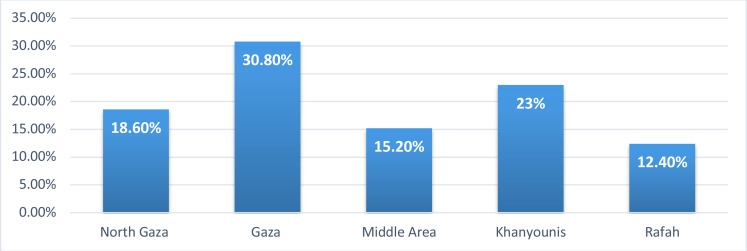
The prevalence of household's food insecurity according to Gaza strip governorates

As shown in Figure 1, the overall prevalence of household's food insecurity was highest in Gaza governorate (30.8%), followed by Khanyounis (23.0%), North Gaza (18.6%), Middle-Area (15.2%) and Rafah (12.4%).

Finally, Table 4 show that people residing in the Rafah governorate were two times more likely to have food insecurity than the North Gaza governorate (crude odds ratio (COR) = 2.02, 95% confidence interval (CI) = [1.02–3.98], P-value < 0.05). In addition, households who had a monthly income of less than 1000 (NIS) were two times more likely to have food insecurity than those who were receiving above 3000 NIS (COR = 2.00, 95% CI = [1.04–2.75], P-value < 0.05). Nonetheless, receiving monthly income of 1000 to 2000 NIS and 2001 to 3000 NIS were a protective variables for food insecurity (COR = 0.10, 95% CI = [0.05–0.19], P-value < 0.05) and (COR = 0.02, 95% CI = [0.01–0.04], P-value < 0.05), respectively. Furthermore, people renting homes were more than two times more likely to have food insecurity than those who own homes (COR = 2.36, 95% CI = [1.39–3.99], P-value < 0.05). Additionally, unemployed people were one time more likely at risk of being food insecure (COR = 1.14, 95% CI = [0.66–1.97], P-value < 0.05).

**Table 4:** The association between the households food security and the demographic, socioeconomic variables (n=1167)

Variables	B (SE)	Crude Odds Ratio (95% CI)	Wald Statistics	P-Value ^a^
Proxy gender				
Male (Ref)	0.19(0.21)	Ref	0.76	0.38
Female		1.20 (0.79–1.85)		
Age (years)	0.009 (0.01)	1.009 (0.99–1.02)	0.98	0.32
Governorates				
North Gaza (Ref)	-0.02 (0.44)	Ref	0.002	0.96
Gaza	0.43 (0.32)	0.97 (0.40–2.35)	1.87	0.17
Middle-Area	0.37 (0.42)	1.55 (0.82–2.90)	0.77	0.37
Khanyounis	0.70 (0.34)	1.45 (0.63–3.37)	4.15	0.04*
Rafah		2.02 (1.02–3.98)		
Living area				
City (Ref)	0.64 (0.39)	Ref	2.60	0.10
Village	-0.27 (0.40)	1.89(0.89–4.13)	0.48	0..48
Camp		0.75 (0.34–1.65)		
Educational level				
Illiterate (Ref)	0.21 (0.98)	Ref	0.04	0.82
Primary	-0.07 (0.83)	1.23(0.18–8.50)	0.008	0.92
Preparatory	0.49(0.81)	0.92(0.18–4.72)	0.36	0.54
Secondary	0.01 (0.81)	1.63 (0.33–8.06)	0.001	0.98
University		1.01 (0.20–4.97)		
Family Members	-0.01 (0.03)	0.98 (0.92–1.05)	0.13	0.71
Monthly income (NIS)a				
Less than 1000	0.70 (0.34)	2.00 (1.04–2.75)	16.83	<0.001*
1000 to 2000	-2.29 (0.33)	0.10(0.05–0.19)	46.82	<0.001*
2001 to 3000	-3.55 (0.27)	0.02 (0.01–0.04)	0.48	0.03*
More than 3000 (Ref)		Ref		
Receive food aids				
Yes (Ref)	-0.24(0.18)	Ref	1.67	0.19
No		0.78(0.54–1.13)		
Homeownership				
Owned (Ref)	0.85 (0.26)	Ref	10.25	0.001*
Rented		2.36(1.39–3.99)		
Work status				
Have work (Ref)	0.13(0.27)	Ref	0.24	0.04*
Do not have work		1.14(0.66–1.97)		
Marital status				
Married (Ref)	-0.87 (0.48)	Ref	3.24	0.07
Divorced	-0.31 (0.55)	0.41(0.16–1.08)	0.32	0.56
Widowed		0.73(0.24–2.14)		

Statistical testing using logistic regression. Ref=Reference category; COR=Denotes crude odds ratio using 95% confidence interval in univariate logistic regression analysis; CI=Confidence interval; B= slop; NIS: New Israeli Shekel.

* Difference is significant at the 0.05 level (2-tailed)

## Discussion

To the best of our knowledge, this is one of the first studies on food security associated factors among the households in the Gaza Strip, Palestine. The main findings of this study indicate that the prevalence of food insecurity was found to be 71.5% out of 1167 included households. The systematic review on measuring and understanding food insecurity in Australia (21) indicated that the Radimer/Cornell food security scale was used in two articles (21, 22). These two papers are based on the same dataset, revealing that 13% of the population is food insecure. Elsahoryi et al. (2020) show that 59.3% of the households in Jordan were food insecure (23). The present study found a greater rate of food insecurity (68.6%) than published by the Food and Agriculture Organization (24). The reported prevalence of food insecurity in our study was high, which may be because of prolonged war, economic stagnation, and limited trade and access to resources in the Gaza strip, combined with high unemployment and poverty rates (25).

In addition, the results revealed that 333 (28.5%) of the households were food secure, 422 (36.2%) had households food insecurity (mild food insecurity), 161 (13.8%) had individual food insecurity (moderate food insecurity), and 251 (21.5%) had child hunger (severe food insecurity). The outcomes of this study were similar to the findings in the Zalilah and Ang study, who reported that among low-income households in Kuala Lumpur, the most experienced level of food insecurity was the household's food insecurity (mild food insecurity) (27.7%) (26). Among the rural New York residents, the most experienced level of food insecurity was the household's food insecurity (25%) (27). The results of our study support these findings. Conversely, the findings in the rural area of Sabak Bernam, which reported that the prevalence of child hunger (severe food insecurity) (34.5%) was the highest among the food-insecure groups (28). As well, the study of Mohamadpour et al. (2012) showed that child hunger was the most typical (40.8%) among the palm-plantation households in Malaysia (29). While in Korea, it was the individual food insecurity (moderate food insecurity) (28.4%) (30). The Radimer/Cornell food security scale rationalizes that children are the last to go hungry in food-insecure households (31). The reason for the higher percentage of household's food insecurity and child hunger than the percentage of individual food insecurity in the Gaza strip has never been discussed and need more studies in the future.

Furthermore, the overall prevalence of household's food insecurity was the highest (30.8%) in the Gaza governorate when compared with the other Gaza strip governorates. The reasons for the higher prevalence of household's food insecurity in the Gaza governorate could be related either to the high number of participants residing in the Gaza or to the fact that most of the food aids are provided to the households located in the Palestinian refugee camps which concentrated at the Middle-Area, North Gaza, and Rafah governorates.

Although the results of this study have shown some expected correlations of demographic and socioeconomic characteristics with household's food insecurity, many previous studies worldwide have emphasized the association between low educational levels and households food insecurity (32, 33). Our findings surprisingly did not support this association and were consistent with the results from Columbia (27). This study did not find a significant association between family size and households food insecurity. This result was consistent with (34), who reported that larger households did not tend to be food-insecure compared to households with small size, and this might be explained by the presence of more adults in the households who contribute financially to the outcome rather than children who do not contribute to the income of the household. In contrast to that, in the United States (32, 35), in the Philippines (36), in Iran (37, 38), and in the UK (39), who found that as the family size increased, the probability of being food-insecure also increased. The non-significant association between the education level and household food insecurity might be attributed to the fact that most of our respondents were moderately educated and the educational level was almost distributed equally between the two groups except for the never been to school.

Our study showed that household income level had been found to influence household's food security in that household with lower incomes is at risk for food insecurity. According to previous studies, food insecurity is more frequent among low-income households (13, 40). This was also corroborated by research by Zalilah and Tham (41) and Henjum et al. (16). Similarly, the more money a family has, the easier it is to get better food, either in terms of quality or quantity (27). Insufficient income among impoverished homes might lead to the inability to provide appropriate meals for family members (29). However, Chan et al. (2020) stated that there is no significant relationship between monthly household income and households food security status (42); as a result, the researchers are compelled to investigate additional possible risk factors that contribute to these respondents' food insecurity (43). Also, Nord and Brent (2002) showed that food insecurity exists among middle- and high-income households (44).

Moreover, the present study showed a statistically significant difference between the employment status of the household head as the unemployed household heads are more likely to be at risk of getting food insecurity. Regarding homeownership, the current study found that people renting homes are more than two times more likely to have food insecurity than those who own homes. This result could be interpreted as the household who cannot afford the price of owing home already living under poor economic status. These findings are in line with those of Frongillo and Zalilah, who found that working-class households had greater food expenditures and also higher levels of food security since they contribute to total family income (45), while our findings were contradicted with Ghanaian and in in Philippines studies, in which the food security status was negatively affected when head works outside the house (36, 46); and the results were also somewhat inconsistent with Canadian study which indicated that employment of the household head is associated with higher rates of restaurant food consumption and higher costs per calorie of home-prepared food (47).

The main limitations of the current study include, social desirability bias may be present in the reporting of food security status. In the context of this investigation, it was not possible to assess food consumption or other markers of nutritional status. Furthermore, the estimations in this study did not take into consideration seasonal variations in food security. According to evidence, food security varies by season (25, 48). Finally, the study's design was cross-sectional, restricting the ability to draw a causal conclusion. The main strength of this study was no indication of selection bias, which included a representative sample of households. A valid and reliable tool (Radimer/Cornell food security scale) was used for determining the households' food security status.

In conclusion, our study demonstrates that food insecurity is highly prevalent (71.5%) in the Gaza strip households. In addition, (36.2%) of the included households had mild food insecurity, (13.8%) had moderate food insecurity, and (21.5%) had severe food insecurity. Furthermore, demographic and socioeconomic factors could contribute to the high prevalence of food insecurity.
